# Ultrasonic Vocalizations in Mice During Exploratory Behavior are Context-Dependent

**DOI:** 10.3389/fnbeh.2015.00316

**Published:** 2015-12-10

**Authors:** Ho-Suk Mun, Tatiana V. Lipina, John C. Roder

**Affiliations:** ^1^Lunenfeld-Tanenbaum Research Institute, Mount Sinai HospitalToronto, ON, Canada; ^2^Department of Molecular Genetics, University of TorontoToronto, ON, Canada; ^3^Federal State Budgetary Scientific Institution, Scientific Research Institute of Physiology and Basic MedicineNovosibirsk, Russia; ^4^Institute of Medical Science, University of TorontoToronto, ON, Canada; ^5^Department of Physiology, University of TorontoToronto, ON, Canada

**Keywords:** ultrasonic vocalizations (USVs), mouse, novelty, exploration, rearings

## Abstract

While rat ultrasonic vocalizations (USVs) are known to vary with anticipation of an aversive vs. positive stimulus, little is known about USVs in adult mice in relation to behaviors. We recorded the calls of adult C57BL/6J male mice under different environmental conditions by exposing mice to both novel and familiar environments that varied in stress intensity through the addition of bright light or shallow water. In general, mouse USVs were significantly more frequent and of longer duration in novel environments. Particularly, mice in dimly-lit novel environments performed more USVs while exhibiting unsupported rearing and walking behavior, and these calls were mostly at high frequency. In contrast, mice exhibited more low frequency USVs when engaging in supported rearing behavior in novel environments. These findings are consistent with data from rats suggesting that low-frequency calls are made under aversive conditions and high-frequency calls occur in non-stressful conditions. Our findings increase understanding of acoustic signals associated with exploratory behaviors relevant to cognitive and motivational aspects of behavior.

## Introduction

Exploration is an essential aspect of behavior, but is also risky and thus avoidance behavior may occur in organisms encountering novel stimuli (Berlyne, 1960). Approach or avoidance is one of the most basic behavioral decisions for animals encountering novel environments. Frequently, approaching new environments is thought to be driven by foraging or mating needs. However, the initial motivation to approach new environments can also be independent of foraging or reproduction, as observed in mice given a choice of novelty vs. food without social cues (Chance and Mead, 1955). A critical question in animal behavior is, what motivates approach behavior and what are the behavioral and neurobiological mechanisms that support such explorative behavior? Indeed, it is currently unknown how to reliably measure motivations or affective states related to approach or avoidance behavior, which is not associated with food or reproduction. Here, we have tried to establish the qualitative and reliable measurement for such phenomenon by analyzing ultrasonic vocalizations (**USVs**).

USVs, above human's hearing range, often reveal a great deal about their general state including motivational or affective states in many species, including songbirds and whales (Wilbrecht and Nottebohm, 2003; Au et al., 2006). Rodents emit USVs that have mostly been studied in pups, in response to maternal isolation, stressors, or rewards (i.e., social interactions; Portfors, 2007). Furthermore, rats communicate with USVs and emit 22 kHz calls during, or in anticipation of threats and 50 kHz calls during, or in anticipation of rewards (See Reviews Knutson et al., 2002; Wöhr and Schwarting, 2013). Although it is generally believed adult mice do not vocalize when encountering novel environments without social factors, recent studies suggest that adult male mice vocalize in a context-dependent manner, including when exposed to novel environments and stressors (Scattoni et al., 2008; Chabout et al., 2012). However, an analysis that includes both USVs and a detailed behavioral repertoire for mice during exploration of novel environments is currently missing in the literature. Moreover, the functional and affective properties of their vocal expressions, in terms of behaviors, have rarely been studied, especially in a non-social context.

The current study attempts to remedy this lack of knowledge using extensive recordings of USVs coupled with behavioral observations that enable us to catalog behavioral correlates for a range of USVs. The experimental set up for recording USVs is mainly focused on separating the relative contributions of the competing tendencies, approach and avoidance (of potentially threatening situations), by manipulating the level of aversive stress (Berlyne, 1960). The “exploratory activity” and other behavior parameters are measured in mice using a novel open field, by manipulating with aversive factors such as the bright light and shallow water (Montgomery, 1955; Dember, 1956; Berlyne, 1960), with simultaneous recording of USVs. Specific aims for this study are: (a) to provide an analysis of USVs that occur in mice exploring environments to ascertain whether they predict emotional states, as shown in rat studies (Wöhr and Schwarting, 2013), (b) to do a simultaneous analysis of exploratory behavioral patterns in mice that display high vs. low call frequencies, (c) to evaluate whether supported and unsupported rearings, which reflect explorative behavior in mice, are associated with certain types of USVs.

## Materials and methods

### Subjects

C57BL/6J male mice (8–10 weeks of age) were housed (three to five per cage) and tested at the Toronto Centre for Phenogenomics (Toronto, Canada) in HEPA-purified, temperature-, and humidity-controlled rooms with 12:12 h light–dark cycle (lights on at 0700 h). Mice received standard chow and water *ad libitum*. Animal use protocols were approved and animals were treated according to the ethical standards defined by the local committee on animal care [Toronto Centre for Phenogenomics (Toronto, Canada)] that conform to the national guidelines (CCAC; http://www.ccac.ca). All efforts were made to minimize animal discomfort and to reduce the number of animals used. To minimize any effects of circadian rhythm on behavioral observations, all experiments were conducted between 0800 and 1100 h. Animal handling was done every day starting from 3 days before the behavioral tests.

### Design of experiments

**Figure 1:**
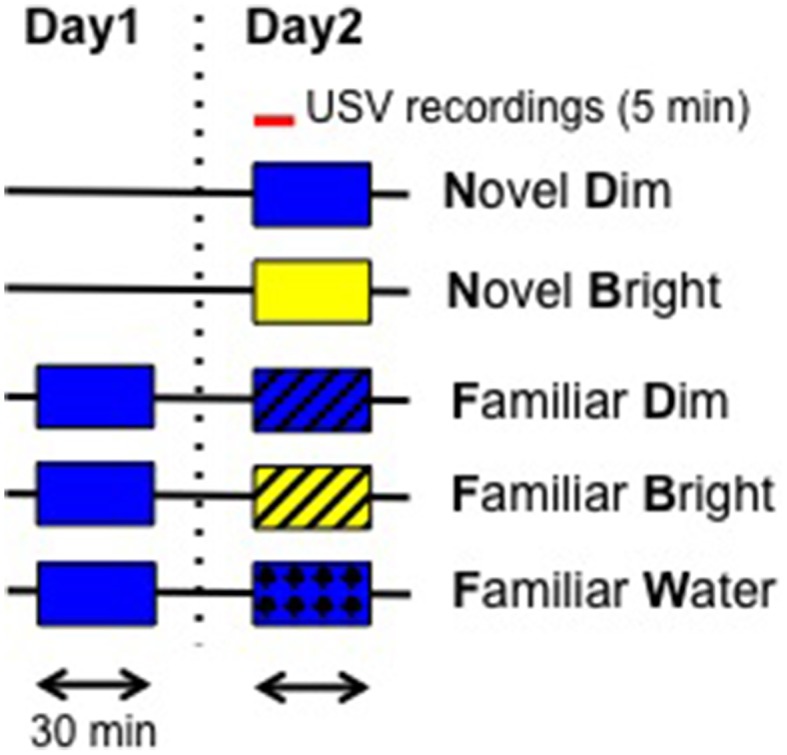
**Schematic representation of experimental procedure**. On day 1, for the familiar groups, mice were pre-exposed to the open field under dim light for 30 min. On the test day, novel groups were exposed to the open field for the first time in two lighting conditions: dim (Blue: 20–40 lux) and bright (Yellow: 400–500 lux). USVs were recorded for the first 5 min. Box without lines represents Novel and Box with the lines represents Familiar conditions. Each arrowed bar represents 30 min. A red bar represents 5 min. USVs: ultrasonic vocalizations.

We considered five treatments. The first four represent a crossed design of the treatments novel vs. familiar with the treatments bright light vs. dim light. We expect exploratory behavior to decrease in these four treatments, from highest to lowest: novel, dim light (ND); novel, bright light (NB); familiar, dim light (FD); familiar bright light (FB) treatments. Bright light is used as an aversive stimulus to simulate a potentially risky environment. As a second aversive stimulus, a fifth treatment was added, placing mice in a previously visited chamber (familiar) that was filled with shallow water (FW; see Figure 1 for an overview of treatment conditions). Handled but naïve to any behavior experiments, C57BL/6J adult male subjects were used. Twelve mice were used in each group. The experimental container was an empty clear Plexiglas chamber (42 × 42 × 42 cm) that was lit from overhead. Two light treatments were used: a dim (20–40 lux) and a bright (400–500 lux) light intensity. For the novel treatments, data were collected from mice exposed to the chamber for the first time, for 30 min in either the dim or bright light condition. For the familiar treatments (under dim or bright light conditions), mice used had been exposed on the previous day to the same chamber under dim lighting (20–40 lux). As a final treatment, water was used as a second stressor, with mice exposed to a novel dimly lit chamber filled with 5 cm of water; this water depth is sufficiently shallow that mice do not need to swim. USVs were recorded (see below) for the first 5 min during each 30 min trial. Animals were returned to their home cages after trials and the entire apparatus was cleaned with 70% ethanol between trials to remove any scent.

### Behavioral recording

Recording and analysis of animal activity behavior was monitored by a video camera fixed on the ceiling 150 cm above the experimental chamber. Behavioral analysis was performed in two ways. First, an experienced observer scored recorded videos using OBSERVER 5.0 software (Noldus Information Technology, Netherlands). The following behaviors were recorded: “immobile”—mouse is passively sitting (>10 s) on one place, with slight movement of the head; “walking”—animal is actively moving; “unsupported rearing”—animal is upright, supported exclusively on hind legs, potentially sniffing the environment; “supported rearing”—animal is upright on hind legs while touching the wall with one or both paws; “grooming”—animal is self-grooming their paws, head and body. Second, the total distance traveled (cm) was analyzed using an automated video tracking system (Ethovision, Noldus, Wageningen, Netherlands).

### Ultrasonic vocalization recording

An UltraSoundGate Condenser Microphone (Avisoft Bioacoustics, Berlin, Germany) was placed 15 cm above the experimental chamber; this was high enough that the receiving angle of the microphone covered the whole area of the test cage. This microphone is sensitive to frequencies of 15–180 kHz with a flat frequency, and was connected via an Avisoft UltraSoundGate 416 USB Audio device (Avisoft Bioacoustics) to a personal computer, where acoustic data were displayed in real time by Avisoft RECORDER USG (Avisoft Bioacoustics). Data were recorded onto the computer with a sampling frequency of 250 kHz, using 1024 points of FFT-length in 16-bit format. For all behavioral conditions USVs were analyzed offline with SASLab Pro (Avisoft Bioacoustics), and a fast Fourier transform was conducted (512 FFT-length, 100% frame, Hamming window and 75% time window overlap). Correspondingly, the spectrograms were produced at a resolution of 488 Hz and 0.512 ms. The number of high frequency (>35 kHz) and low frequency (20–35 kHz) calls was filtered out automatically using SASLab Pro. The following acoustic features were counted by Pulse train analysis: (1) call duration—mean duration of a single USV, and (2) mean peak frequency, expressed as peak frequency at maximal amplitude. To compare USVs with behavior we synchronized audio and video files by performing a “clap” with fingers in the field of the camera to time-match video and audio files. In the audio files, we cut the information before this sound, and in the video files we selected the exact time frame of this event and started behavioral scorings at this time-point. This manual synchronization permitted us to link those behaviors described above, with USVs elicited at the time of the behavioral event.

### Statistical analyses

Behavioral and USVs were analyzed by Two-way analysis of variance (ANOVA) followed by Bonferroni *post-hoc* testing. Acoustic features are presented in box and whisker plots with median, upper and lower quartiles and maximum and minimum values. Behavioral data in figures are expressed as mean ± standard error of the mean (SEM). Differences were considered statistically significant at *p* < 0.05.

## Results

### USVs during exploration of novel environment

**Figure 2:**
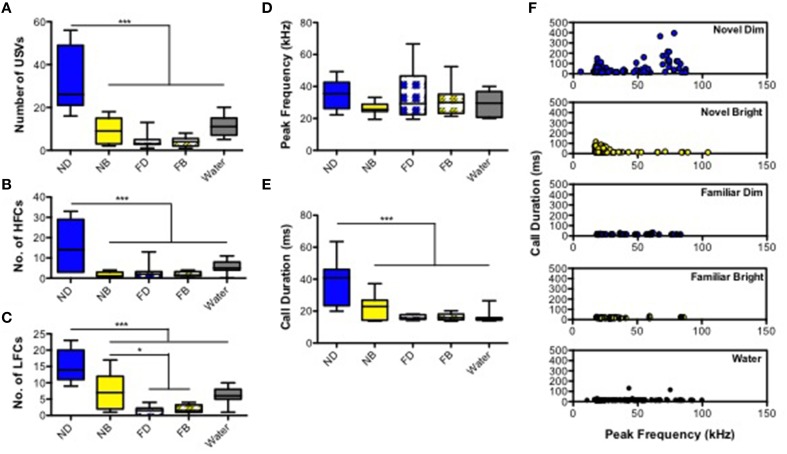
**Ultrasonic vocalizations (USVs) in adult male mice**. Box and whisker plots represent **(A)** total number of USVs, **(B)** number of high frequency calls (>35 kHz), and **(C)** number of low frequency calls (20–35 kHz) were displayed. For the acoustic features, **(D)** mean peak frequency, **(E)** call duration, and **(F)** distribution of calls with frequency and call durations were shown peak in each environments, including ND, novel dim; NB, novel bright; FD, familiar dim; FB, familiar bright; and water. ^*^P < 0.05, ^***^P < 0.001 (Bonferroni *post-hoc* analysis; Two-way ANOVA).

We first quantified and analyzed the number and features of USVs emitted in the five different treatment conditions: ND, NB, FD, FB, and FW (Figure 1). Two mice, each from FD and FW conditions, failed to utter any calls and therefore were excluded from further analysis. The number of USVs emitted by adult male mice differed significantly among treatments [Figure 2A; Supplementary Table 1A; *F*_(4, 48)_ = 24.16, *p* < 0.0001]. *Post-hoc* comparisons showed that mice in the ND condition emitted significantly more calls than mice in all other conditions. We then, examined the number of high and low frequency calls in the five treatments. Both high and low frequency call number varied by treatment [high frequency calls: Figure 2B; Supplementary Table 1B; *F*_(4, 48)_ = 12.93, *p* < 0.0001; low frequency calls: Figure 2C; Supplementary Table 1C; *F*_(4, 48)_ = 23.37]. Mice in the ND condition emitted significantly more calls at both frequencies than mice in all other conditions (Figures 2B,C; Supplementary Tables 1B,C). However, the number of calls was significantly greater for mice in the NB condition compared to the FD and FB conditions only for low frequency calls (Figures 2B,C; Supplementary Table 1C). Mice in the NB condition also had the largest percentage of low frequency calls (out of total number of all calls) at 78.93 ± 5.38%, compared to ND (53.64 ± 6.17%), FD (50 ± 13.15%), FB (52.29 ± 8.91%), and FW (57.05 ± 6.91%).

There was no main effect of experimental groups on peak frequency [Figure 2D; Supplementary Table 1D; *F*_(4, 48)_ = 2.23, *p* = 0.079]. There did appear to be a trend in which mice in ND, FD, and FB conditions emitted somewhat higher mean peak frequency USVs (34.93 ± 2.79, 34.91 ± 5.47, and 31.04 ± 3.16 kHz, respectively), and mice in NB and FW conditions tended to emit lower mean peak frequency USVs (26.01 ± 1.16 and 28.74 ± 2.34 kHz, respectively; Figure 2D). Mean call duration differed significantly among treatments [*F*_(4, 48)_ = 14.737; *p* < 0.0001; Figure 2E; Supplementary Table 1E]; call duration was significantly longer in the ND group than in all other groups (Figure 2E). We then plotted the distribution of individual USV call durations by peak frequency for each treatment separately (Figure 2F). This illustrates well the effect of experimental conditions, where calls of high frequencies USV have a longer duration in the ND treatment relative to the others (Figure 2E).

### Acoustic features in behaviors

**Figure 3:**
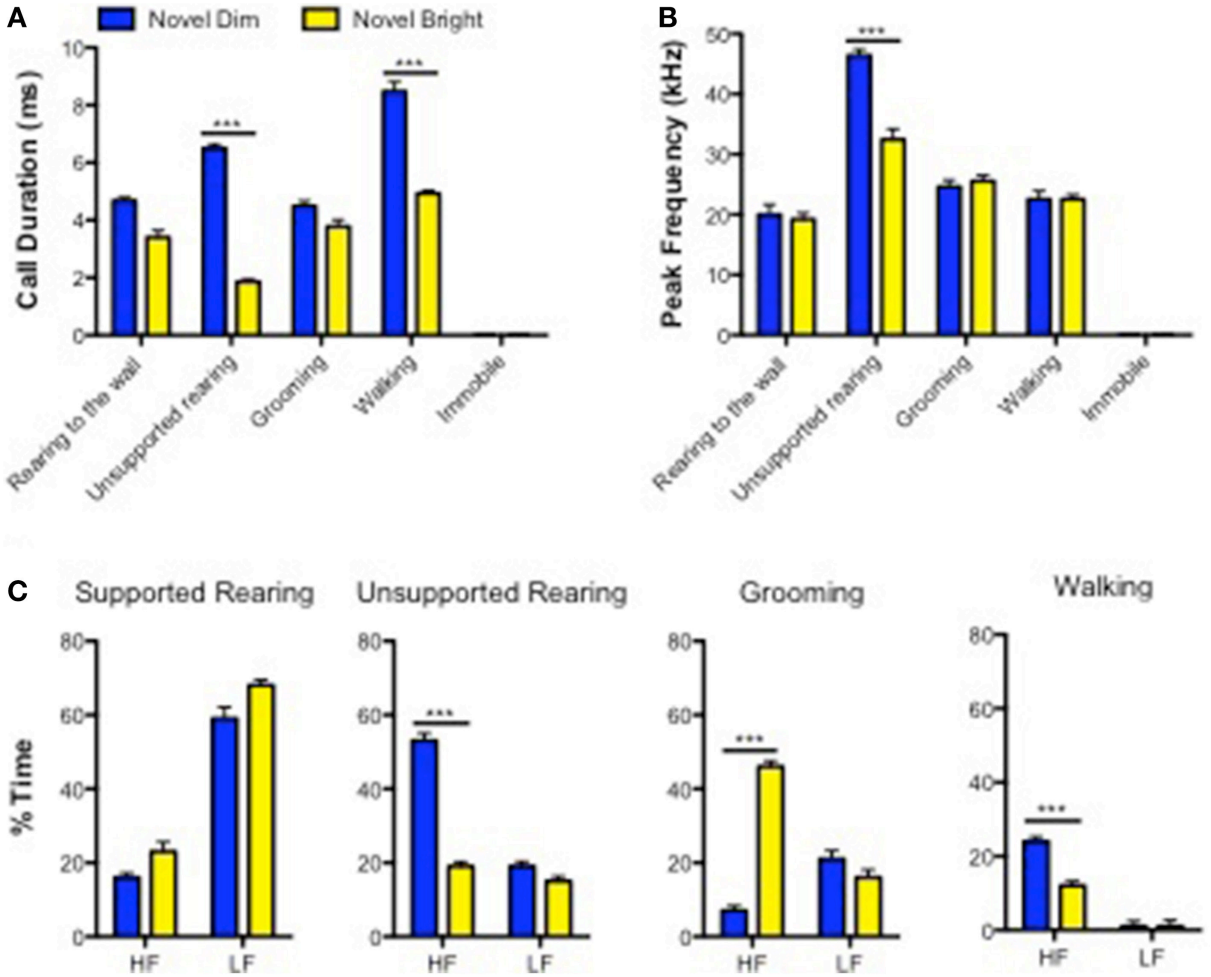
**Acoustic characteristics of calls emitted in behaviors in novel dim and novel bright contexts**. **(A)**Calls durations and **(B)** Peak Frequency of calls in all conditions. **(C)** Percent time mice engaged in the behaviors “supported rearing,” “unsupported rearing,” “self-grooming,” and “walking” spend making “high-frequency (HF)” and “low-frequency (LF)” calls. Data are presented as means ± SEM. ^***^p < 0.0001—in comparison with “Novel Bright” condition (Bonferroni *post-hoc* analysis; Two-way ANOVA).

In order to characterize the relationship between acoustic features of USVs and behaviors, we analyzed the duration and peak frequency of USV calls emitted while an animal was engaging into certain types of behaviors. We used only ND and NB conditions for this, based on our findings that mice demonstrated the most enriched USVs' repertoire under these conditions (Figure 2). There was a significant interaction between treatment group and behavior on call duration [*F*_(3, 302)_ = 17.03, *p* < 0.0001; Figure 3A; Supplementary Table 2A]. *Post-hoc* comparisons showed that while performing unsupported rearing or walking behaviors, call duration was longer in ND compared to NB (Figure 3A; Supplementary Table 2A). Similarly, for USV peak frequency, a significant interaction was present between treatment and behavior [*F*_(3, 302)_ = 6.577; *p* = 0.0002], with only mice displaying unsupported rearing showing an effect of treatment, where peak frequency was higher for mice in the ND treatment (Figure 3B; Supplementary Table 2B). Notably, mice did not emit any USVs while not moving (Figures 3A,B).

Next, we analyzed high and low frequency calls in the same ND and NB experimental groups made while performing four most preferable behaviors: walking, self-grooming, unsupported and supported rearings. A Two-way ANOVA revealed an interaction effect between three behavioral paramters (the exception was supported rearing) and percent time mice spent giving either high frequency or low-frequency USVs (Supplementary Table 2C). For the supported rearing, only the main effect of frequency was significant: low frequency USVs were more frequently elicited than high-frequency USVs for both treatment conditions: ND and NB (Figure 3C). Mice of the ND group produced more high-frequency calls when they demonstrated unsupported rearings and walking, relative to the NB group (53 vs. 19%, respectively; Figure 3C). During self-grooming behavior this pattern was reversed and mice (46%) produced more high-frequency calls under the NB (46 vs. 7%; Figure 3C) than under the ND condition.

## Discussion

In this study, we explored mouse USVs in non-social contexts, manipulating the level of aversive condition by adding a bright light or shallow water to the experimental chamber. We show that mouse vocalizations vary in a context and/or behavior dependent manner. While exploration of a novel environment with a potentially aversive factor, a bright light, appears to differentially influence mouse' vocal behavior compared to novel environments without aversive factors, adult male mice emitted fewer calls in familiar conditions relative to novel condition. More specifically, mice were engaged in unsupported rearing behavior with emission of high frequency calls during exploring a novel dim light environment. In contrast, animals demonstrated avoidance, such as unsupported rearing, in the more stressful situation (novel bright light), which was coupled with low frequency calls. A detailed characterization of mouse vocalization in association with particular behavioral performance offers a unique opportunity to decipher vocalizations in mice.

Notably data on whether mice can emit USVs in a non-social context is severely lacking, thereafter still debatable. One report showed that mice would not produce USVs during exposure to aversive stimulation such as physical restraint or electric shock (Portfors, 2007). However, another study recorded USVs in adult mice in non-social contexts such as exploration of a novel environment or restraint stress (Chabout et al., 2012), showing USVs in both novel and aversive environments. Our study revealed that adult male mice are able to emit calls during exploration in both familiar and novel environments, as well as brightly-lit novel environments.

Moreover, mice differentially emitted USVs depending on the environment, although the total number of calls we saw emitted during exploration was generally lower than the number observed during social interactions (Holy and Guo, 2005; Chabout et al., 2012). As expected, adult male mice exposed to a familiar environment elicited very few calls, regardless of the light treatment. This may indicate that USVs reflect physiological conditions, given that mice are typically less aroused and active after habituation (Harris, 1943). However, the habituation effect does not explain the few calls made in familiar bright environment, since we assumed that bright light could elicit aversive states, therefore, the number of low frequency call should have increased. To unveil the possibility of the bright light being not enough to elicit aversive states, we used another stronger aversive factor, water. However, we still found low number of USVs similar to that of brightly-lit familiar conditions. This finding is in line with a study that found fewer USV calls in restrained stress compared to in novel environments (Ko et al., 2005; Chabout et al., 2012). Notably, exploration of novelty under less stressful conditions (dim light) triggered the largest number of USVs in mice; this condition presumably reflects pure exploration, without aversion, since the impact of stress was minimized. The bright light given in the novel environment significantly reduced number of USVs which supports the idea that aversive states reduces USVs. Overall, our findings indicate that USVs might serve as a robust index of an animal's response to the stress.

The detailed characterization of acoustic signals in association with exploratory behavior reveals that acoustic features of mouse USVs are distinctive depending on context. In rats, acoustic calls are divided into high frequency calls (50 kHz) emitted during anticipation of reward or approach behavior and low frequency calls (22 kHz) emitted during anticipation of punishment or avoidance behavior (Knutson et al., 2002). Recent studies characterized USVs in mice, and focused on vocal repertoire while excluding USVs < 25 kHz (Holy and Guo, 2005; Wang et al., 2008). In our study, by recording the whole spectrum of USVs, we found that mice in the “bright light group” (higher stress) emitted USVs at a mean frequency of 26.01 ± 1.16 kHz. These results are similar to recent findings that adult male mice may emit low frequency 30 kHz (26–36 kHz) calls when stressors are present (Ko et al., 2005) and emit high frequency 40 kHz calls with social rewards (Chabout et al., 2012). The distinctive call patterns may be aligned with the 50 kHz reward calls and 22 kHz aversive calls as shown for rodents, since bright light is aversive *per se* to mice and exploration under dim light is likely self-rewarding for mice.

While other studies scored a selection of behaviors, including USV emission, in a given time period and showed correlation between the two, we analyzed simultaneously the different behavioral patterns accompanying the USVs. USVs were emitted in conjunction with most behavioral categories involving movements, and were not detected in immobile states. These findings agree with other studies, where high vocalization rates correlated with high levels of locomotor activity in rats (Fu and Brudzynski, 1994) and in mouse' pups (Branchi et al., 2004). Behavioral patterns also depended on the context. Mice from the “bright light group” spent more time in the corner and were more likely to rear against the wall, reflecting escape-oriented behavior, whereas the “dim light group” showed more exploratory behavior, such as unsupported rearing and time spent in the chamber's center. Further, our analysis revealed that mice were more likely to show unsupported rearing behavior when eliciting high frequency calls and more likely to rear against a wall, when emitting low frequency calls. Given that high-frequency calls are associated with pleasure (Chabout et al., 2012), this association between the exploratory unsupported rearing behavior and high frequency calls suggests that exploration with minimal stress levels is likely self-rewarding for mice.

We propose here first insight into how emotional and motivational individual states on novelty exploration are connected with emission of USVs in adult male mice. The widespread application of USVs in characterizing neuropsychiatric mouse models has been hampered by the use of pups (immature brain) and a lack of comprehensive studies in mice (Portfors, 2007). We argue that this framework is important for exploring mouse models of neuropsychiatric disorders (e.g., depression, autism, Rett syndrome) by characteristics of USVs during exploration of novel environments because failure to be engaged in such activity may reflect consequences of the profound defects of sensory-motor and cognitive functions, so deficits in sensory-motor or cognitive functions may contribute to the reduced exploratory behavior in children with autism spectrum disorder (Pierce and Courchesne, 2001) and in elderly people with Alzheimer's disease (Daffner et al., 2001). Conversely, the increased novelty seeking properties might contribute to such mental disorders as substance abuse (See Review, Bardo et al., 1996) or manic episodes of bipolar disorder (Regier et al., 2013). The association we found in this study, between certain types of USVs and behavioral exploratory patterns, demonstrate a novel way to study USVs in animal models of neuropsychiatric disease. This approach will help us to understand the acoustic capacities of mice and may ultimately allow us to select novel vocal phenotypes for animal models of mental disorders, to which various behavioral designs and multiple genetic mouse lines can be applied.

### Conflict of interest statement

The authors declare that the research was conducted in the absence of any commercial or financial relationships that could be construed as a potential conflict of interest.
