# Supplementary material for: Ultrasonic Vocalizations in Mice During Exploratory Behavior are Context-Dependent
Source: Front Behav Neurosci. 2015 Dec 10;9:316. doi: 10.3389/fnbeh.2015.00316 (PMC4674556; doi:10.3389/fnbeh.2015.00316)
Supplement: Supplementary file 1 [file Table1.PDF]

Ultrasonic Vocalizations in Mice During Exploratory Behavior are Context-Dependent.

Ho-suk Mun*, Tatiana V. Lipina, John C. Roder

*** Correspondence:** Ho-suk Mun; mun@lunenfeld.ca

Supplementary Table 1. Details of statistical results for group effects by one-way ANOVA and for paired comparisons of total number of calls for each call types in the five different conditions by Bonferroni post-hoc test.

A **Total number of Calls** (*Group Effect F(4,48)=24.16, p<0.001*)

	NB (n=11)	FD (n=10)	FB (n=10)	Water (n=11)
ND (n=11)	t=6.934, p<0.001	t=8.250, p<0.001	t=8.341, p<0.001	t=6.311, p<0.001
NB (n=11)	-	t=1.483, p=1.000	t=1.574, p=1.000	6=0.622, p=1.000
FD (n=10)	-	-	t=0.089, p=1.000	t=2.091, p=0.419
FB (n=10)	-	-	-	t=2.182, p=0.340

B **High Frequency Calls** (*Group Effect F(4,48)=12.93, p<0.001*)

	NB (n=11)	FD (n=10)	FB (n=10)	Water (n=11)
ND (n=11)	t=6.106, p<0.001	t=5.522, p<0.001	t=5.848, p<0.001	t=4.551, p<0.001
NB (n=11)	-	t=0.437, p=1.000	t=0.111, p=1.000	t=1.555, p=1.000
FD (n=10)	-	-	t=0.318, p=1.000	t=1.081, p=1.000
FB (n=10)	-	-	-	t=1.406, p=1.000

C **Low Frequency Calls** (*Group Effect F(4,48)=23.37, p<0.001*)

	NB (n=11)	FD (n=10)	FB (n=10)	Water (n=11)
ND (n=11)	t=4.726, p<0.001	t=8.408, p<0.001	t=8.099, p<0.001	t=5.706, p<0.001
NB (n=11)	-	t=3.796, p=0.004	t=3.487, p=0.016	t=0.979, p=1.000
FD (n=10)	-	-	t=0.302, p=1.000	t=2.840, p=0.072
FB (n=10)	-	-	-	t=2.531, p=0.202

D **Peak Frequency** (*Group Effect F(4,48)=2.23, p=0.079*)

	NB (n=11)	FD (n=10)	FB (n=10)	Water (n=11)
ND (n=11)	t=2.118, p=0.33	t=0.005, p=1.000	t=0.875, p=0.925	t=1.470, p=1.000
NB (n=11)	-	t=2.005, p=0.219	t=1.134, p=1.000	t=0.648, p=1.000
FD (n=10)	-	-	t=0.830, p=0.653	t=1.390, p=1.000
FB (n=10)	-	-	-	t=0.519, p=1.000

E **Call Duration** (*Group Effect F(4,48)=14.737, p<0.001*)

	NB (n=11)	FD (n=10)	FB (n=10)	Water (n=11)
ND (n=11)	t=4.279, p=0.001	t=5.973, p<0.001	t=5.903, p<0.001	t=6.060, p<0.001
NB (n=11)	-	t=1.913, p=0.531	t=1.844, p=1.000	t=1.781, p=0.455
FD (n=10)	-	-	t=0.066, p=1.000	t=0.223, p=1.000
FB (n=10)	-	-	-	t=0.154, p=1.000
