# Supplementary material for: Ultrasonic Vocalizations in Mice During Exploratory Behavior are Context-Dependent
Source: Front Behav Neurosci. 2015 Dec 10;9:316. doi: 10.3389/fnbeh.2015.00316 (PMC4674556; doi:10.3389/fnbeh.2015.00316)
Supplement: Supplementary file 2 [file Table2.PDF]

Ultrasonic Vocalizations in Mice During Exploratory Behavior are Context-Dependent.

Ho-suk Mun*, Tatiana V. Lipina, John C. Roder

*** Correspondence:** Ho-suk Mun; mun@lunenfeld.ca

Supplemental Table 2. Related to Figure 3. Details of statistical results for group effects by two-way ANOVA and for paired comparisons of peak frequency (**A**) and call duration (**B**) in the Novel Dim and Novel Bright groups by Bonferroni post-hoc tests.

A

**2-way ANOVA**	Interaction	F(3,302)=17.03	p<0.0001
	Group	F(1,302)=127.2	p<0.0001
	Behavior	F(3,302)=32.89	p<0.0001
**Bonferroni posttest (Novel Dim vs. Novel Bright)**			
Supported Rearing		t=4.85	p<0.001
Unsupported rearing		t=10.04	p<0.001
Grooming		t=1.45	P>0.05
Walking		t=9.99	p<0.001
Immobile		t=0.00	P>0.05

B

**2-way ANOVA**	Interaction	F(3,302)=6,577	p=0.0002
	Group	F(1,302)=6.252	p=0.0129
	Behavior	F(3,302)=41.15	p<0.0001
**Bonferroni posttest (Novel Dim vs. Novel Bright)**			
Supported Rearing		t=0.49	p>0.05
Unsupported rearing		t=4.36	p<0.001
Grooming		t=0.29	p>0.05

	Walking	t=0.01	p>0.05
	Immobile	t=0.00	p>0.05

C **Supported Rearing**

**2-way ANOVA**	Interaction	F(1,40)=0.08	p=0.7707
	Group	F(1,40)=5.51	p=0.0239
	Frequency	F(1,40)=166.7	p<0.0001
**Bonferroni posttest (Novel Dim v. Novel Bright)**			
	High Frequency	t=1.45	p>0.05
	Low Frequency	t=1.86	p>0.05

Unsupported Rearing

**2-way ANOVA**	Interaction	F(1,40)=89.81	p<0.0001
	Group	F(1,40)=144.1	p<0.0001
	Frequency	F(1,40)=144.1	p<0.0001
**Bonferroni posttest (Novel Dim v. Novel Bright)**			
	High Frequency	t=19.15	p<0.001
	Low Frequency	t=1.78	p>0.05

Grooming

**2-way ANOVA**	Interaction	F(1,40)=136.1	p<0.0001
	Group	F(1,40)=81.29	p<0.0001
	Frequency	F(1,40)=18.00	p<0.0001
**Bonferroni posttest (Novel Dim v. Novel Bright)**			
	High Frequency	t=14.63	p<0.001
	Low Frequency	t=1.88	p>0.05

**Walking**			
**2-way ANOVA**	Interaction	F(1,40)=16.9	p<0.0001
	Group	F(1,40)=16.9	p<0.0001
	Frequency	F(1,40)=135.7	p<0.0001
**Bonferroni posttest (Novel Dim v. Novel Bright)**			
High Frequency		t=5.81	p<0.001
Low Frequency		t=0.00	p>0.05
